# Knowledge, Attitudes and Practice of Healthcare Providers, Healthcare Regulatory Practitioners and Patients Toward Biosimilars in China: Insights From a Nationwide Survey

**DOI:** 10.3389/fphar.2022.876503

**Published:** 2022-06-02

**Authors:** Yang Hu, Zaiwei Song, Dan Jiang, Lin Zhuo, Yinchu Cheng, Rongsheng Zhao

**Affiliations:** ^1^ Department of Pharmacy, Peking University Third Hospital, Beijing, China; ^2^ Institute for Drug Evaluation, Peking University Health Science Center, Beijing, China; ^3^ Therapeutic Drug Monitoring and Clinical Toxicology Center, Peking University, Beijing, China; ^4^ Department of Pharmacy Administration and Clinical Pharmacy, School of Pharmaceutical Sciences, Peking University, Beijing, China; ^5^ Research Center of Clinical Epidemiology, Peking University Third Hospital, Beijing, China

**Keywords:** biosimilars, knowledge, attitudes, practice, China

## Abstract

**Objective:** With increasing numbers of biosimilars entering the market or in the approval pipeline in China, understanding the current awareness and attitudes of biosimilars still remains the first step to promote uptake. This study aims to investigate the knowledge, attitudes and practices (KAP) of multiple stakeholders toward biosimilars, including healthcare providers (HCPs), healthcare regulatory practitioners and patients, and to provide practical information for future uptake of biosimilars in China.

**Methods:** This nationwide cross-sectional online survey was conducted in mainland China. The questionnaire with a high level of reliability and validity was designed based on previous studies and clinical questions in the Clinical Practice Guideline for Clinical Application of Biosimilars. Logistic regression model was employed to identify possible impact factors, and Spearman’s rank correlation test was used to identify the correlation between knowledge and attitudes. Chi-squared test was used to compare the differences between different stakeholders.

**Results:** Overall, 599 valid respondents were recruited, of whom 77.63%, 7.01% and 15.36% were HCPs, healthcare regulatory practitioners and patients, respectively. A total of 504 respondents who had heard of biosimilars were included in the KAP analysis. 76.70% of HCPs, 90.24% of healthcare regulatory practitioners and 50.98% of patients had good knowledge about the definition, while less familiarity with the development process and regulations on interchangeability and indication extrapolation was found in the former two groups. For attitudes toward biosimilars, an overall lack of positivity was shown, as only 18.20% HCPs, 14.63% healthcare regulatory practitioners and 23.53% patients were classified as having positive attitudes. More specifically, most respondents were positive about the influence of payment policy on the uptake of biosimilars, but they showed a neutral attitude toward the clinical medication and interchangeability of biosimilars. Efficacy, safety, immunogenicity, interchangeability and indication extrapolation are major concerns when utilizing biosimilars. Regarding practice, our study showed an inadequate utilization of biosimilars in China. Several further suggestions on the regulation of biosimilars were proposed by healthcare regulatory practitioners.

**Conclusions:** There is still plenty of room for improvement of knowledge, attitudes and practice toward biosimilars among multiple stakeholders in China, which can be improved through high-quality real world evidence, educational programs and other effective measures directed towards barriers.

## Introduction

A biosimilar product is a biologic that is highly similar but not identical to an already licensed reference product in terms of quality, safety, and efficacy (Kirchhoff et al., 2017). In 2006, the first biosimilar medicine, Omnitrope^®^ (biosimilar recombinant human growth hormone [rhGH]; Sandoz, Kundl, Austria), was approved in Europe by the European Medicines Agency (EMA) (Pfaffle et al., 2020). Since February 2022, 33, 69 and 18 biosimilars have received approval by the Food and Drug Administration (FDA) (Food and Drug Administration, 2021), EMA (European Medicines Agency, 2022) and China’s National Medical Products Administration (NMPA), respectively. In addition, an increasing number of newly developed biosimilars are expected to hit the global market and involve malignant tumors, the immune system, the blood system, the digestive system, the skin and connective tissue and other diseases.

Currently, global spending on medicine continues to grow. Of the increasing spending, biologics are responsible for $120 billion or 37% of net drug spending in the United States, and since 2014, for 93% of the overall growth in total spending (Lexchin, 2020). Partly due to the costly manufacturing facilities and the large investment in research and development, biologics are extremely expensive and represent a major financial burden. It has been well recognized that biosimilars provide lower-cost alternatives to their reference medicines and confer significant cost-saving advantages, yielding the potential to mitigate rising drug costs. Thus, in theory, biosimilars can expand the treatment options available to patients and increase the accessibility of therapeutic and supportive care to patients. For example, it has been proposed that the sustainability of cancer care worldwide can potentially be improved through the use of safe and effective biosimilars (Giuliani et al., 2019).

However, despite the potential for reducing care costs and improving patient access, the uptake of biosimilars has been slow. Limited biosimilar growth has contributed to a time-consuming approval process, regulatory challenges, legal issues, payer policy disparities, patients’ willingness and barriers from healthcare providers (HCP) (Leonard et al., 2019). Among HCPs, uptake is sometimes limited by uncertainties in reduced efficacy or safety in real clinical practice, immunogenicity concerns, and a lack of understanding of the concept of extrapolation and interchangeability (Giuliani et al., 2019; Sarnola et al., 2020). Successful introduction of biosimilars into the clinic will depend in part on HCPs’ understanding, promoting, prescribing, and using biosimilars in clinical practice, and patients’ willingness and confidence.

As increasing numbers of biosimilars seek market entry or are in the approval pipeline, understanding current attitudes and awareness of biosimilars is the first step to assess the need for biosimilar education, promote utilization, and, ultimately, help drive down biologic therapy costs. Previous surveys have assessed the knowledge and beliefs of physicians or pharmacists or patients regarding biosimilars in the United States (US) (Cohen et al., 2017; Gibofsky and McCabe, 2021), Britain (Chapman et al., 2017), France (Beck et al., 2017; Frantzen et al., 2019), Poland (Pawlowska et al., 2019), Russia (Karateev and Belokoneva, 2019), Brazil (Garcia et al., 2021), Pakistan (Shakeel et al., 2020), etc. Studies concluded that familiarity and attitudes toward biosimilars may vary across different regions, as respondents from countries where a more mature market of biosimilars existed tended to have a better level of knowledge and more positive attitudes. Most physicians or pharmacists remain cautious about using these agents, citing limited familiarity with biosimilars, contributing to safety and efficacy concerns and limited biosimilar prescribing. However, as the world’s second-largest market for pharmaceuticals and the fastest emerging market for the sector (Qu et al., 2021), data looking at Chinese HCPs or patients are still lacking. Besides, from a global perspective, there still exists a gap in healthcare regulatory practitioners’ perspectives on biosimilars.

Thus, to elucidate current practices and hindering factors of biosimilars uptake in China, we investigated multiple stakeholders’ knowledge, attitudes, and practices (KAP) toward biosimilars by conducting a nationwide cross-sectional online survey, including HCPs (physicians, clinical pharmacists, and nurses), healthcare regulatory practitioners, and patients. We aimed to provide practical guiding information for the future development of biosimilars in China.

## Methods

### Study Design

The convenience sampling approach was used to recruit HCPs, healthcare regulatory practitioners, and patients by disseminating the recruitment notice with the online questionnaire link attached in the WeChat groups. The notice contained a brief introduction, including the study background, objective, procedures, voluntary nature of participation, declarations of anonymity and confidentiality, and notes for completing the questionnaire. All physicians, clinical pharmacists, nurses, practitioners of drug regulation, medical administration, medical insurance and pharmacoeconomics, and patients from mainland China who consented to the survey were considered eligible. Two similar questions in the questionnaire were set as logic check items, which were used to assess the quality of the collected questionnaires. We excluded answer sheets that 1) missed key information such as the specialty of the HCPs and 2) collected contradictory answers judged by the logic check items.

### Questionnaire Design and Pilot Survey

The questionnaire was designed based on previous studies and the clinical questions in the Clinical Practice Guideline for Clinical Application of Biosimilars (Hu et al., 2021), which was developed and justified after three rounds of consultation with senior clinical pharmacists and methodologists. The main content of the questionnaire for different groups of respondents was similar, with a few items tailored according to the characteristics of different occupations. For patients, plain language was used to ensure a better understanding.

The questionnaire consisted of four parts: demographic information, knowledge, attitudes, and practice. For healthcare professionals and healthcare regulatory practitioners, demographic variables included occupations (only for HCPs), province/region, gender, age, education background, hospital classifications (only for HCPs), job title, work seniority, specialty (for HCPs: hematology, oncology, rheumatology, gastroenterology and others; for healthcare regulatory practitioners: drug regulation, medical insurance, medical administration and pharmacoeconomics), overall familiarity with biosimilars (the questionnaire would be closed directly to respondents who never heard of biosimilars) and information source. For patients, income was investigated instead of information on their occupation. The knowledge part included four questions for HCPs and healthcare regulatory practitioners on biosimilars’ definition, research and development process, indication extrapolation regulations in China, and interchangeability regulations in China. For patients, only 1 question on biosimilars’ definition was asked. The attitude part consisted of three sections regarding attitudes (A1) toward medication of biosimilars, (A2) toward interchangeability of biosimilars, and (A3) toward the influence of payment policy on the uptake of biosimilars. The attitudes were measured on a 5-point Likert scale, with one score standing for strongly disagree to five for strongly agree (Singleton et al., 2021). The questions in the practice part varied by the identity of the respondents, with 10 for physicians, 7 for clinical pharmacists, 4 for nurses, 5 for healthcare regulatory practitioners and 3 for patients. The experience of using biosimilars was asked among HCPs and patients, and future suggestions on the regulation of biosimilars were investigated in healthcare regulatory practitioners.

A pilot survey was conducted to confirm the feasibility and reliability of the questionnaire in a small sample of multidisciplinary HCPs (*n* = 21), including oncologists, hematologists, rheumatologists, gastroenterologists, clinical pharmacists and nurses. Cronbach’s alpha of A1, A2, A3 and all questions in the attitude part was respectively 0.905, 0.910, 0.908 and 0.940, indicating a high level of internal consistency and reliability (Taber, 2018). The content validity of the questionnaire was evaluated by combining the feedback of the pilot survey and the experts’ opinions.

### Sample Size Calculation

The sample size was calculated according to the Cochran (1977) formula (Cochran, 1977), where n is the sample size;

$$n=\frac{{\left(Z_{\alpha /2}\right)}^{2}p\left(1-p\right)}{\Delta^{2}}$$

$Z_{\alpha /2}$ is the confidence level of 95% (standard deviation 1.96); p is the awareness rate in this study, and we assumed the expected awareness rate is 50% as it would lead to the largest sample size; △ is the accuracy level, which was set as 5% (0.05) in our study. In addition, an invalid estimating rate of 20% was considered. Therefore, the sample size in this study was estimated to be 480.

### Statistical Analysis

The statistical analyses were performed with SPSS software version 26.0 (IBM Corp., Armonk, NY, United States). A multiple imputation approach was used for missing data. For quantitative data following a normal distribution, we calculated the mean with standard deviations (mean ± SD) and used Pearson’s rank correlation test to identify the possible correlation between variables. For non-normally distributed data, we calculated the median with interquartile range [median (IQR)] and used Spearman’s rank correlation test to identify the correlation among variables. We calculated the frequency and proportion (%) for qualitative data and used the chi-squared test to compare the differences between the groups. The level of knowledge, attitudes and practice were summarized and classified. A correct rate of answers above 50% was considered “qualified”, while below 50% was considered “unqualified”. A score of attitudes above four was regarded as “positive,” while below four was deemed “not positive.” Only those who had heard of biosimilars were included in the KAP analysis.

A logistic regression model was used to identify independent factors of knowledge and attitudes among different respondents. First, univariate analysis was performed to identify possible factors. Variables with statistical significance as well as those determined by reading relevant literature and combining clinical experience, including occupation (only for HCPs), gender, job title, education background, work seniority (or age for patients), specialty (or patients’ disease classification), and family income (only for patients), were then included in the multivariate logistic regression using the Enter method. All statistical analyses were two-tailed, and *p* < 0.05 was considered statistically significant.

### Ethical Approval

All procedures performed in this study involving human participants followed the Declaration of Helsinki (as revised in 2013). The study was approved by the institutional ethics board of Peking University Third Hospital (NO. IRB00006761-M2021443). The online voluntary and anonymous questionnaire secured the participants’ confidentiality and did not collect any identity-exposing information of the participants.

## Results

### Basic Information

**FIGURE 1:**
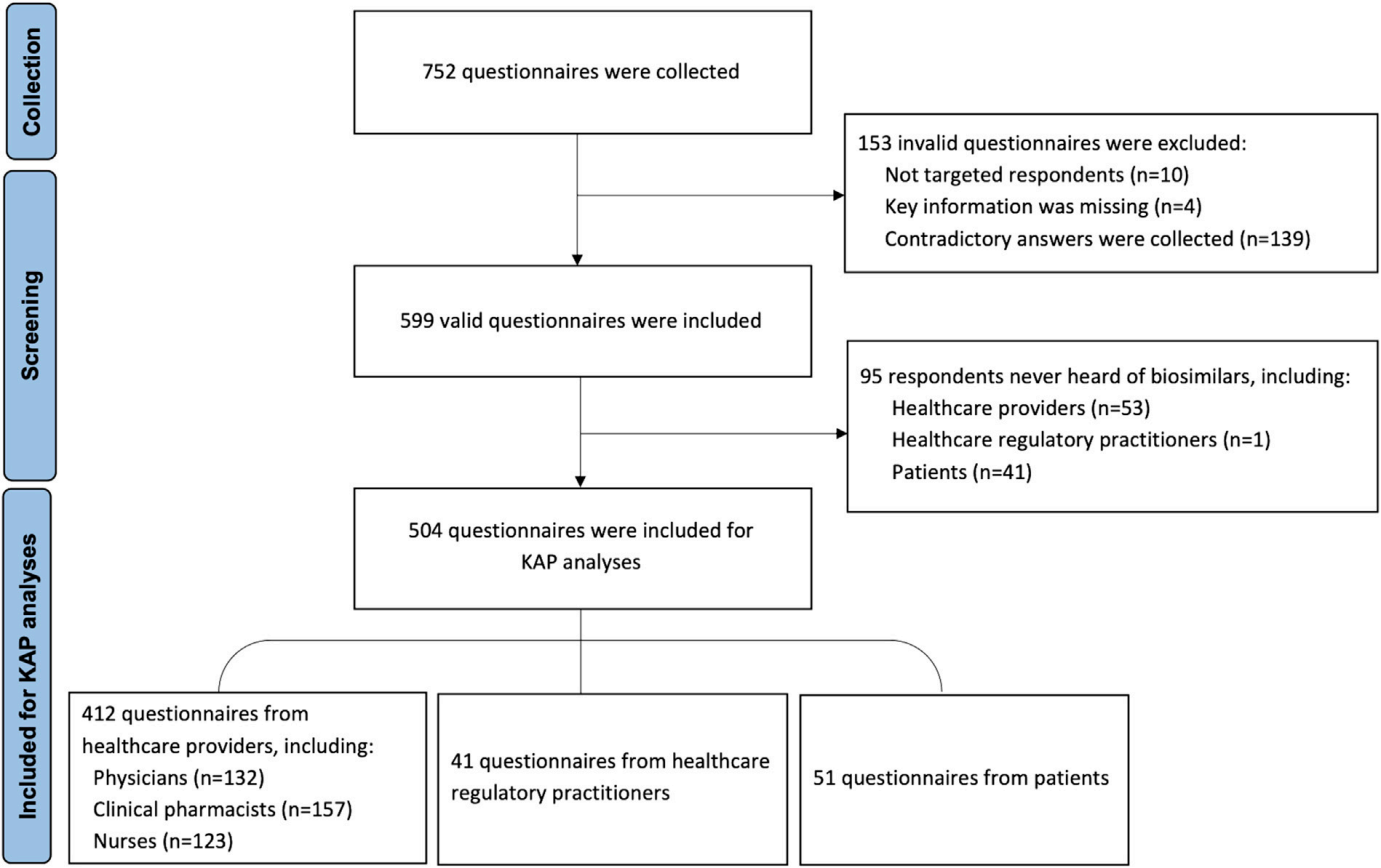
Flow diagram of including and excluding the questionnaires.

A total of 752 respondents participated in the survey, of which 153 were considered invalid and were excluded. 504 of 599 valid respondents were included in the KAP analysis. The procedure for including and excluding the respondents is shown in Figure 1. Among all valid respondents, 465 (77.63%) were HCPs, 42 (7.01%) were healthcare regulatory practitioners, and 92 (15.36%) were patients who came from various regions of mainland China. Demographic characteristics are shown in Table 1.

**TABLE 1:** Demographic characteristics of respondents.

Characteristics	Subgroup	465 healthcare providers [n (%)]	42 healthcare regulatory practitioners [n (%)]	92 patients [n (%)]
Occupation	Physician	144 (30.97)	-^a^	-
	Clinical pharmacist	166 (35.70)	-	-
	Nurse	155 (33.33)	-	-
Region	North China	210 (45.16)	1 (2.38)	29 (31.52)
	Northeast China	49 (10.54)	18 (42.86)	10 (10.87)
	East China	61 (13.12)	14 (33.33)	20 (21.74)
	Central China	17 (3.66)	4 (9.52)	3 (3.26)
	South China	9 (1.94)	5 (2.38)	0
	Southwest China	91 (19.57)	0	27 (29.35)
	Northwest China	28 (6.02)	7 (9.52)	3 (3.26)
Gender	Male	101 (21.72)	18 (42.86)	36 (39.13)
	Female	364 (72.28)	24 (57.14)	56 (60.87)
Age (year)	<30	117 (25.16)	1 (2.38)	19 (20.66)
	30–39	248 (53.33)	32 (76.19)	28 (30.43)
	40–49	83 (17.85)	7 (16.67)	16 (17.39)
	≥50	17 (3.66)	2 (4.76)	29 (31.52)
Hospital classification	Tertiary	455 (97.85)	-	-
	Secondary	9 (1.94)	-	-
	Primary	1 (0.22)	-	-
Job title	Junior or below	162 (34.84)	2 (4.76)	-
	Intermediate	202 (43.44)	18 (42.86)	-
	Senior	101 (21.72)	22 (52.38)	-
Education background	Below bachelor	26 (5.59)	0	46 (50.00)
	Bachelor	172 (36.99)	4 (9.52)	34 (36.96)
	Master	170 (36.56)	14 (33.33)	10 (10.87)
	Doctor	97 (20.86)	24 (57.14)	2 (2.17)
Work seniority (year)	1–5	126 (27.10)	12 (28.57)	-
	6–10	154 (33.12)	14 (33.33)	-
	11–15	90 (19.35)	12 (28.57)	-
	16–20	48 (10.32)	2 (4.76)	-
	≥21	47 (10.11)	2 (4.76)	-
Specialty (Disease classification)	Drug regulation	-	9 (21.43)	-
	Medical insurance	-	8 (19.05)	-
	Medical administration	-	4 (9.52)	-
	Pharmacoeconomics	-	21 (50.00)	-
	Hematology	33 (7.10)	-	8 (8.70)
	Oncology	241 (51.83)	-	41 (44.57)
	Rheumatology	18 (3.87)	-	12 (13.04)
	Gastroenterology	22 (4.73)	-	13 (14.13)
	Others^b^	151 (32.47)	-	18 (19.57)
Family income (yuan)	<5,000	-	-	29 (31.52)
	5,000–8,000	-	-	12 (13.04)
	8,000–12,000	-	-	19 (20.65)
	12,000–15,000	-	-	17 (18.48)
	>15,000	-	-	15 (16.30)
Familiarity with biosimilars	Never heard of it	53 (11.40)	1 (2.38)	41 (44.57)
	Only heard of it, but not familiar	105 (22.58)	6 (14.29)	32 (34.78)
	Somewhat familiar	164 (35.27)	18 (42.86)	16 (17.39)
	Familiar	130 (27.96)	14 (33.33)	3 (3.26)
	Very familiar	13 (2.80)	3 (7.14)	0
Information source^c^ (Multiple-select)	Guideline or consensus of biopharmaceuticals	291 (62.58)	26 (61.90)	-
	Consensus or technical guidance of biosimilars	207 (44.52)	21 (50.00)	-
	Academic articles	243 (52.26)	21 (50.00)	-
	Academic conferences	248 (53.33)	26 (61.90)	-
	Internet	141 (30.32)	9 (21.43)	-
	Introduction from pharmaceutical companies	176 (37.85)	13 (30.95)	8 (8.70)
	News media	81 (17.42)	13 (30.95)	22 (23.91)
	Introduction from medical staff	-	-	40 (43.48)
	Science lectures	-	-	12 (13.04)

a Choices not aiming for corresponding respondents.

b Other specialties include endocrinology, dermatology, geriatrics, pediatrics, gynecology, stomatology, infectious disease, traditional Chinese medicine, cardiology, neurology, orthopedics, etc.

c Only respondents who had heard of biosimilars selected the information source.

Among the included HCPs, 35.70% were clinical pharmacists, 72.28% were females, 97.85% were employed in tertiary hospitals, 51.83% specialized in oncology and 72.90% had been working for more than five years. 53 of 465 HCPs had not heard of biosimilars, and the main information source of biosimilars among the rest of the HCPs was guidelines or consensus of biopharmaceuticals, followed by academic articles and conferences.

Among the included healthcare regulatory practitioners, 57.14% were females, 50.00% had expertise in pharmacoeconomics and 71.43% had been working for more than five years. Only one practitioner had not heard of biosimilars. The main information source of biosimilars among healthcare regulatory practitioners were guidelines or consensuses of biopharmaceuticals and academic conferences.

60.87% of patients who participated in this survey were female, and most held a bachelor’s degree or below. 44.57% of the patients suffered from tumors, and 41 of 92 had no idea of biosimilars. The main information source for patients who had heard of biosimilars was an introduction from medical staff.

### Knowledge of Biosimilars Among Different Respondents

Overall, the number of correct answers was 2 [1] (median [IQR]) among HCPs, and 56.07% of them were classified as qualified. The results of the knowledge part varied among different occupational subgroups. The number of correct answers was 2 [2] in physicians and clinical pharmacists, while it was only 1 [1] in nurses. 90 (68.18%) physicians, 98 (62.42%) clinical pharmacists and 43 (34.96%) nurses were classified as qualified. Most HCPs had the correct understanding of the definition of biosimilars (K1). However, less familiarity with the research and development process, indication extrapolation regulations in China and interchangeability regulations in China (K2-4) were found in medical staff, as the correct answer rates of these three questions were 30.58%, 50.73% and 15.78%, respectively.

Healthcare regulatory practitioners performed slightly better in the knowledge of biosimilars than HCPs. The number of correct answers was 3 [3], and 29 (70.73%) healthcare regulatory practitioners were classified as qualified. The correct answer rate of K1 on biosimilars’ definition was 90.24%. Fewer healthcare regulatory practitioners answered correctly in terms of research and development processes, indication extrapolation regulations in China and interchangeability regulations in China (K2-4).

The definition of biosimilars (K1) was the only question to measure patients’ understanding of biosimilars, and 26 of 51 patients (50.98%) answered correctly. The correct answer rates of each question in the knowledge part among different respondents are shown in Table 2.

**TABLE 2:** Results of Knowledge among different respondents.

Item	Correct answer rate (%)					
	Healthcare providers (*n* = 412)	Physicians (*n* = 132)	Clinical pharmacists (*n* = 157)	Nurses (*n* = 123)	Healthcare regulatory practitioners (*n* = 41)	Patients (*n* = 51)
K1	76.70	81.82	82.17	64.23	90.24	50.98
K2	30.58	39.39	38.85	10.57	58.54	-
K3	50.73	55.30	59.24	34.96	60.98	-
K4	15.78	18.18	18.47	9.76	41.46	-

K1-Which of the statements best describes your understanding of biosimilars: (wrong) Biosimilars are identical to reference products in terms of structure, biological activity, safety and efficacy; (correct) Biosimilars are similar, but not identical to reference products in terms of structure, biological activity, safety and efficacy; (wrong) Biosimilars should have superior safety and efficacy profile to reference products; (wrong) None of the above options are correct.

K2-Is the proportion of each step in the development process (including *in vitro* studies, non-clinical studies, clinical pharmacological studies and clinical trials) of biosimilars the same as that of reference products: (correct) No.

K3-Is it permitted in China that biosimilars can be automatically approved for all indications of the reference products based on clinical studies in just one of the indications: (correct) No.

K4-Is there a clear definition of “interchangeable biosimilar product” in the review and registration process in China: (correct) No.

### Attitudes Toward Biosimilars Among Different Respondents

Overall, regardless of their identities, respondents showed a neutral attitude toward the medication of biosimilars (A1) and interchangeability of biosimilars (A2). However, regarding the influence of payment policy on the uptake of biosimilars (A3), most respondents held a positive attitude.

**FIGURE 2:**
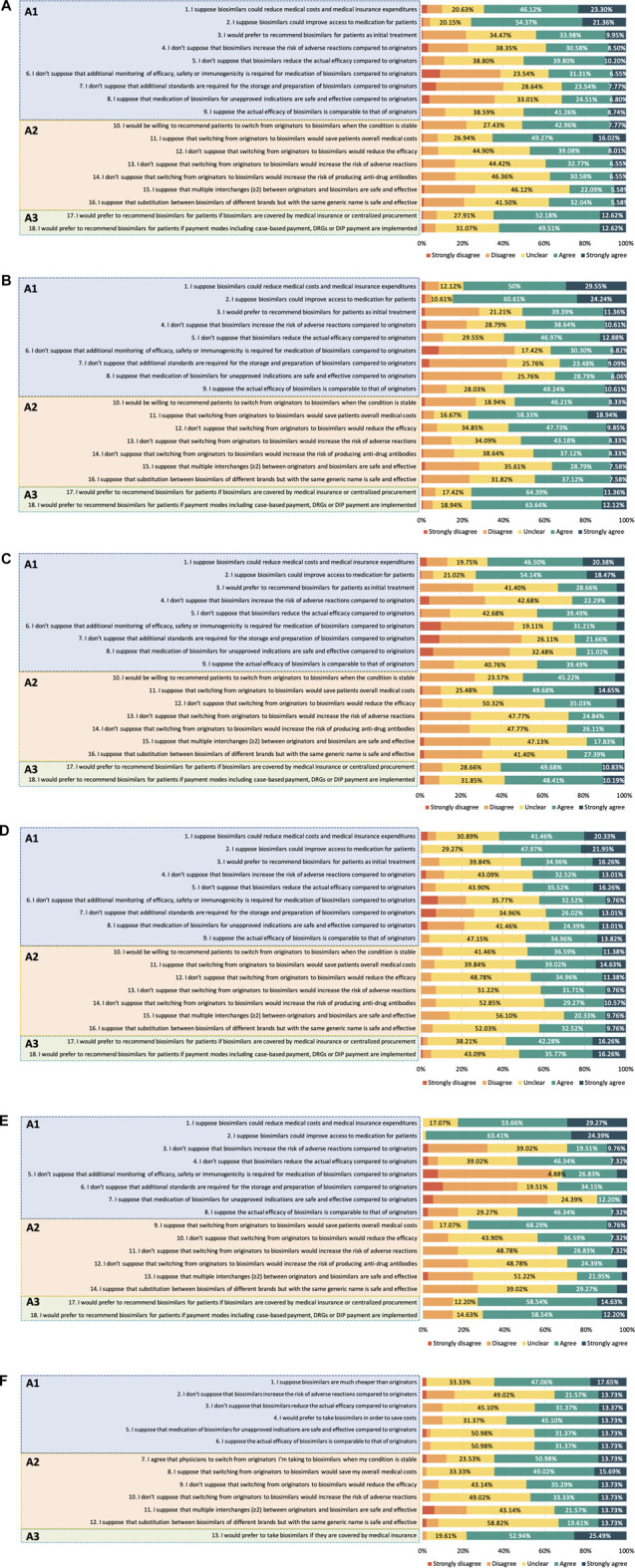
Continued.

Among HCPs, 75 (18.20%) were classified as having positive attitudes. Their attitudes toward biosimilars are shown in Figure 2A. The scores of A1, A2 and A3 were 3.22 [0.89] (median [IQR]), 3.14 [0.86], and 4.00 [1.00], respectively. Most HCPs held positive attitudes toward the economic effect of biosimilars. The medical staff was more concerned about biosimilar safety profiles than efficacy. Only 39.08% of them thought biosimilars would not increase the risk of adverse reactions compared to the reference products. As for medication of biosimilars for unapproved indications, 31.31% of HCPs showed a positive attitude. When asked about their attitudes toward the interchangeability of biosimilars, 50.73% of HCPs reported they would be willing to recommend patients to switch from originators to biosimilars when the condition is stable. However, most HCPs felt unclear or worried about the efficacy (52.91%), safety (60.68%), and immunogenicity (62.87%) after switching to biosimilars.

Attitudes to biosimilars were not alike across different occupational subgroups among HCPs. Figures 2B–D shows the results of the attitudes of physicians, clinical pharmacists and nurses. In terms of medication of biosimilars and the interchangeability of biosimilars, clinical pharmacists held a relatively conservative attitude compared with physicians and nurses. The overall score of A1 was 3.33 [0.78] in physicians, 3.00 [0.78] in clinical pharmacists and 3.33 [1.00] in nurses. The overall scores of A2 among physicians, clinical pharmacists and nurses were 3.42 [1.00], 3.00 [0.86], and 3.29 [1.00], respectively. Attitudes to the influence of payment policy on the uptake of biosimilars converged to positive among different HCPs, as the overall scores of A3 were 4.00 [0.50], 4.00 [1.00], and 4.00 [1.00] in physicians, clinical pharmacists and nurses, respectively.

Among healthcare regulatory practitioners, only 6 (14.63%) were classified as having positive attitudes. The scores of A1, A2 and A3 were 3.25 [0.63], 3.17 [0.92], and 4.00 [1.00], respectively. The results of their attitudes are shown in Figure 2E. Similar to the HCPs, a small proportion (29.27%) of healthcare regulatory practitioners showed a positive attitude toward biosimilar safety and supposed biosimilars would not have a higher risk of adverse reactions than their reference counterparts. 68.30% of them suggested that it is necessary to monitor biosimilar efficacy, safety, or immunogenicity compared with reference products. Only 14.64% felt positive about the medication of biosimilars for unapproved indications. A majority of healthcare regulatory practitioners showed uncertainty or negative attitudes about efficacy (56.10%), safety (65.85%), and immunogenicity (70.73%) after switching to biosimilars from originators.

Among patients, 12 (23.53%) of 51 showed an overall attitude as positive. The scores of A1, A2, and A3 were 3.33 [1.00], 3.33 [1.00], and 4.00 [1.00], respectively. As shown in Figure 2F, most patients felt unclear about the biosimilars’ efficacy (49.02%) and safety (45.10%), while 58.83% reported they would prefer to take biosimilars to save medical costs. In terms of interchangeability of biosimilars, 64.71% of patients agreed to switch from originators to biosimilars when their condition was stable, whereas fewer had positive attitudes toward efficacy (49.02%) and safety (47.06%) after switching.

### Current Practice Status of Biosimilars Among Different Respondents

**FIGURE 3:**
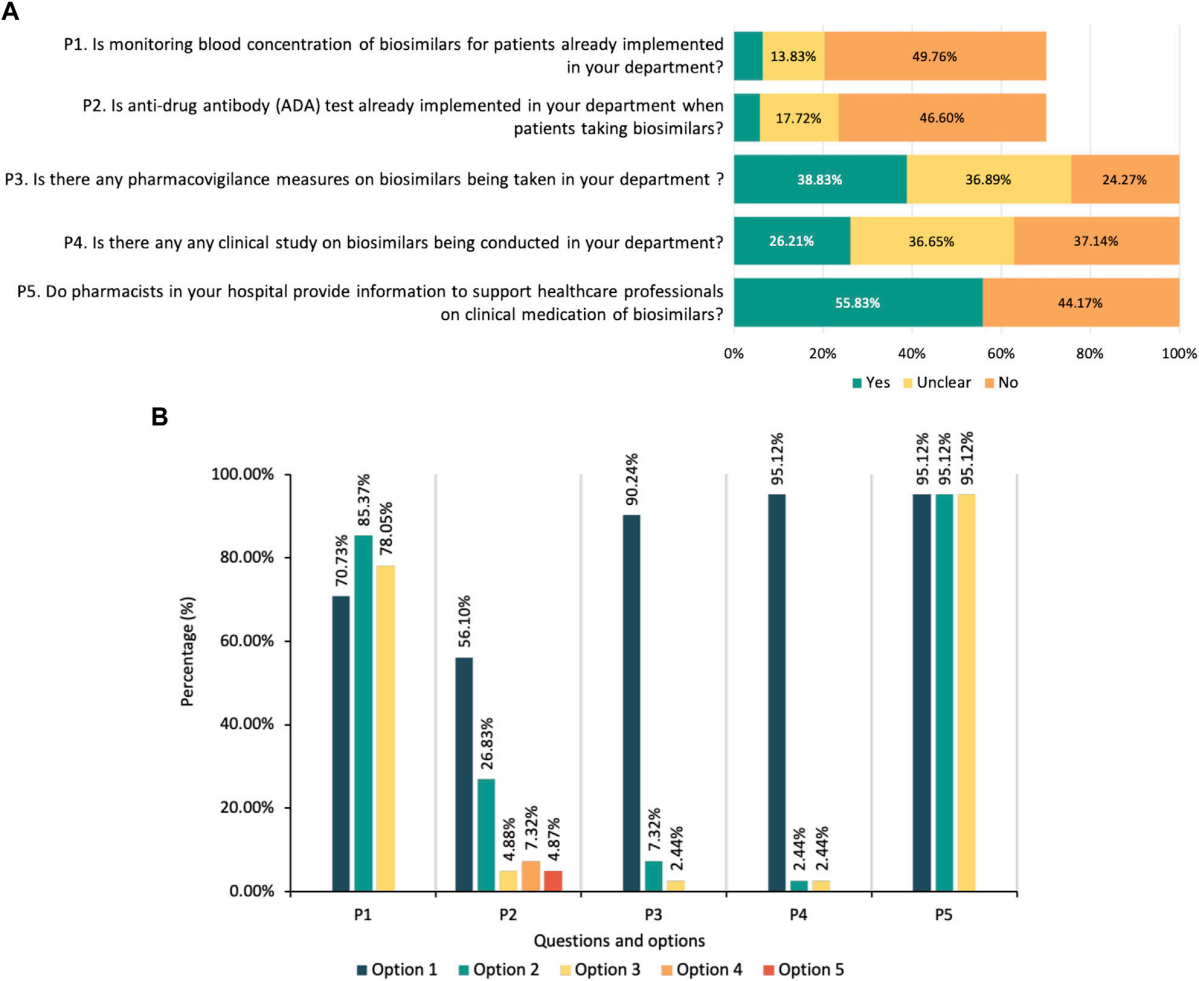
**(A)** Current practice status of biosimilars among healthcare providers. Note: P1 and P2 are questions only for physicians and clinical pharmacists. **(B)** Suggestions on the regulation of biosimilars from healthcare regulatory practitioners. Note: P1-What do you think needs to be improved in the registration and review process of biosimilars in China (multiple-select): (Option 1) a more definite review process needs to be established; (Option 2) the technical guidance for development and evaluation of biosimilars needs to be updated; (Option 3) requirements for registration of biosimilars needs to be more clarified. P2-Under which of the following circumstances do you think extrapolation of biosimilars’ indication can be considered: (Option 1) the biosimilar has the same mechanism of action for the indication in the head-to-head comparison study as for the proposed extrapolated indication; (Option 2) the pathophysiological mechanisms are similar for the indication in the head-to-head comparison study and the proposed extrapolated indication; (Option 3) pharmacokinetics of the biosimilar are similar in different populations; (Option 4) Immunogenicity of the biosimilar is similar in different populations; (Option 5) Other opinions, including the proposed extrapolated indication should be supported by clinical evidence, or agree with all above options. P3-Is it necessary to put forward the concept of “interchangeable biosimilars” in China: (Option 1) yes; (Option 2) no; (Option 3) unclear. P4-Is it necessary to set regulations on clinical switching of biosimilars in China: (Option 1) yes; (Option 2) no; (Option 3) unclear. P5-What pharmacovigilance measures do you think should be strengthened in the clinical medication of biosimilars: (Option 1) developing a risk management plan for the clinical medication of biosimilars; (Option 2) Recording detailed information of biosimilars, including brand name, batch and dosage, etc., to ensure traceability; (Option 3) Strengthening the monitoring and reporting of adverse reactions of biosimilars.

Questions in the practice part were tailored for respondents according to their identity. HCPs and patients were asked about their experience of using biosimilars. Overall, 42.96% of HCPs had enrolled in the clinical practice of biosimilars, including 76 physicians who had prescribed biosimilars, 59 pharmacists and 42 nurses. Among the physicians, 46 (34.85%) had experienced a non-medical switch between originators and biosimilars. 38 (28.79%) reported that they had prescribed biosimilars for unapproved indications based on guidelines or consensuses of the reference products. When prescribing the biosimilars, 74 (56.06%) would obtain informed consent from patients (55 reported signing an informed consent form was necessary) and made some explanations about biosimilars to the patients, including the efficacy and potential risks of biosimilars, the influence of biosimilars on medical costs, and the definition of biosimilars. The current status of implementing therapeutic drug monitoring, pharmacovigilance and conducting clinical research on biosimilars was also investigated, and the results are shown in Figure 3A.

Among patients, 15 of 51 (29.41%) reported they had taken biosimilars, and 4 of them had the experience of switching from an originator to a biosimilar. After switching, one patient reported that she had experienced an adverse reaction that had not occurred before. For healthcare regulatory practitioners, future suggestions on the regulation of biosimilars were investigated (Figure 3B).

### Impact Factors and Correlation Analysis of Knowledge and Attitude

We found that HCPs’ education background and specialty were possible independent factors of their knowledge. Multivariate logistic regression showed that HCPs with a master’s degree had better knowledge than those with a bachelor’s degree (OR = 2.33, 95% CI = 1.20–4.54, *p* = 0.01). The medical staff of specialties not covering the medication of biosimilars took poor command of the knowledge part compared with oncologists (OR = 0.32, 95% CI = 0.21–0.51, *p* < 0.01), while there was no significant difference between hematologists, rheumatologists, gastroenterologists, and oncologists. In terms of attitudes, gender might be an independent factor, as multivariate analysis showed that males tended to be more positive than females (OR = 2.43, 95% CI = 1.27–4.66, *p* < 0.01).

Due to the relatively limited sample size of healthcare regulatory practitioners and patients, multivariate logistic regression was not suitable for the analysis of those respondents. Univariate analyses showed that the possible impact factors of knowledge for healthcare regulatory practitioners were specialty, gender, and education background. Medical insurance experts had poorer knowledge than those specializing in pharmacoeconomics (OR = 0.11, 95% CI = 0.02–0.70, *p* = 0.02). Males seemed to have a better understanding than their female counterparts (OR = 15.58, 95% CI = 1.77–137.36, *p* = 0.01), and holding bachelor’s degrees might result in poorer knowledge than holding doctoral degrees (OR = 0.07, 95% CI = 0.01–0.86, *p* = 0.04). However, the results were opposite in terms of attitudes. The possible impact factor of attitudes was education background, and healthcare regulatory practitioners with bachelor’s degrees seemed to be more positive than those with doctoral degrees (OR = 31.50, 95% CI = 2.14–463.14).

For patients, no variable seemed to affect their knowledge, and the only possible impact factor of attitude was educational background. Compared with patients holding degrees lower than bachelor’s, those with bachelor’s degrees were less positive about biosimilars (OR = 0.09, 95% CI = 0.01–0.79, *p* = 0.03).

The correlation between knowledge and attitude was explored with Spearman’s rank correlation test. The results showed that knowledge was negatively related to attitudes among HCPs (r_s_ = -0.129, *p* < 0.01). There was no relation between knowledge and attitudes among healthcare regulatory practitioners (r_s_ = -0.039, *p* = 0.81) and patients (r_s_ = -0.167, *p* = 0.24).

### Comparison of Knowledge and Attitudes Among Different Respondents

The chi-squared (χ^2^) test was performed to compare the knowledge and attitudes among HCPs, healthcare regulatory practitioners and patients. In terms of knowledge of biosimilar definition, both HCPs and healthcare regulatory practitioners performed better than patients (HCPs vs. patients: 76.69% vs. 50.98%, χ^2^ = 15.55, *p* < 0.01; healthcare regulatory practitioners vs. patients: 90.24% vs. 50.98%, χ^2^ = 16.232, *p* < 0.01). There was no significant difference in knowledge between HCPs and healthcare regulatory practitioners (χ^2^ = 3.28, *p* = 0.07). Regarding attitudes, no significant difference was found between the three groups of respondents (HCPs vs. patients: χ^2^ = 0.84, *p* = 0.36; healthcare regulatory practitioners vs. patients: χ^2^ = 1.14, *p* = 0.29; HCPs vs. healthcare regulatory practitioners: χ^2^ = 0.32, *p* = 0.57).

## Discussion

As a lower-cost alternative to originators, biosimilars have been expected to expand patient access to life-saving medications for difficult-to-treat disorders and to ultimately reduce medical expenditure (Cohen et al., 2017). However, despite the potential financial advantages, the uptake of biosimilars in China has still been stunted. Given the rapid development of biosimilars, understanding current awareness and attitudes is the first step to assess the necessity for biosimilar education and promote utilization. Although previous studies have found that familiarity and attitudes toward biosimilars may vary across different regions, as the second-largest pharma market around the globe (Qu et al., 2021), there is no study focusing on assessing the KAP of biosimilars among related stakeholders in mainland China. Thus, with the aim of filling this gap between current knowledge and practice, we pay more attention to multiple stakeholders in mainland China in this study, including HCPs (physicians, clinical pharmacists, and nurses), healthcare regulatory practitioners and patients.

### Overall Findings in Knowledge About Biosimilars

Generally, most respondents have heard of biosimilars, and the knowledge level of biosimilars varied with the respondents’ identities. Among HCPs, most of them (76.70%) showed appropriate knowledge regarding the definition of biosimilars, which is similar to United Kingdom HCPs(Chapman et al., 2017). However, only a few HCPs were acquainted with the research and development process and regulations on indication extrapolation and interchangeability. More specifically, in occupational subgroups, the correct answer rates of the definition of biosimilars were 81.82%, 82.17% and 64.23% in physicians, clinical pharmacists and nurses, respectively. For physicians, our results showed that Chinese physicians had a comparable level of knowledge about what biosimilars were to United States specialty physicians (Cohen et al., 2017) and European prescribers (Giuliani et al., 2019) and a higher level in comparison with Russian physicians (Karateev and Belokoneva, 2019). For clinical pharmacists, their knowledge of biosimilar definition is comparable with studies in other countries, such as Pakistan (Shakeel et al., 2020), France (Beck et al., 2017) and Poland (Pawlowska et al., 2019). Nurses showed a relatively poorer overall knowledge of biosimilars compared with physicians and clinical pharmacists.

Among patients, less adequate understanding of biosimilars was found in comparison with HCPs (*p* < 0.01). Only 50.98% of the patients chose the correct definition, which showed a similar inadequate level of knowledge about biosimilars to several published studies (Peyrin-Biroulet et al., 2017; Azevedo et al., 2018; Vandenplas et al., 2021a). This emphasizes an existing knowledge gap and the further need for tailored education for patients.

Healthcare regulatory practitioners showed a marginally better level of knowledge about biosimilars than HCPs, whereas the difference did not reach statistical significance. Most healthcare regulatory practitioners (90.24%) took good command of the definition of biosimilars. More than half of them were familiar with the research and development process and regulations on indication extrapolation. Although a larger proportion of healthcare regulatory practitioners correctly answered the current regulations on interchangeability of biosimilars than HCPs (41.46% vs. 15.78%), it was still the most unacquainted item for Chinese healthcare regulatory practitioners.

### Overall Findings in Attitudes Toward Biosimilars

An overall lack of positivity was found among all respondents. More specifically, respondents showed a positive attitude to the influence of payment policy on the use of biosimilars, but they held a neutral attitude to the clinical medication and interchangeability of biosimilars with concerns from several aspects. Interestingly, after analyzing the correlation between knowledge and attitude, we found that medical staff with a higher level of knowledge tended to be less positive toward biosimilars. As biosimilars are inherently different from chemically manufactured generics due to their molecular size, structure, and the complexity of their development (Oza et al., 2019), it is understandable that HCPs with better awareness of these properties might wear a more conservative and vigilant attitude.

The results of our study showed that most respondents were positive about the financial advantages of biosimilars, which was consistent with various studies in other countries (Chapman et al., 2017; Peyrin-Biroulet et al., 2017; Azevedo et al., 2018; Park et al., 2019). Patients may directly benefit from the cost savings of biosimilars through lower insurance premiums and lower out-of-pocket costs (Kvien et al., 2022). In addition, payment policy was considered as a key driver for biosimilar uptake. The majority of HCPs and healthcare regulatory practitioners supported biosimilars utilization if they were covered by the Chinese centralized drug procurement program (Wang et al., 2021). It is shown that this aforementioned policy promoted the use of generics and had positive effects on drug price cut and medication burden reduction (Yang et al., 2022). Similarly, uptake of biosimilars and control of medical costs might also gain benefit if biosimilars could be selected in centralized procurement program. Moreover, several provider payment modes have been implemented in some regions in China to control health expenditures, such as case-based payment (Wang et al., 2019), diagnosis-related groups (DRGs) payment (Duan et al., 2021) and diagnosis-intervention packet (DIP) payment (Lai et al., 2022). With more pilot implementation of these payment modes over the coming years, biosimilars might be considered as a cost-effective option to optimize the sustainability of healthcare systems.

However, concerns about biosimilars that may hinder uptake still exist. A large percentage of respondents were unsure or concerned about the efficacy and safety of biosimilars, which was similar to studies conducted in Europe and the United States(Barsell et al., 2017; Beck et al., 2017; O'Callaghan et al., 2017; Peyrin-Biroulet et al., 2017; Aladul et al., 2019; Frantzen et al., 2019). In terms of medication for extrapolated indications, most respondents showed neutral or even negative attitudes, which was consistent with HCPs in Canada and patients in Portugue (Peyrin-Biroulet et al., 2017; Azevedo et al., 2018). Whereas contradictory result was shown in a previous study where most prescribers felt comfortable using a biosimilar in an extrapolated indication (Giuliani et al., 2019). In terms of the interchangeability of biosimilars, an overall positive attitude was observed among patients toward switching from originators to biosimilars when their condition was stable, and half of the HCPs agreed with the choice. Nonetheless, concerns over safety and efficacy after switching remained among most respondents, and similar results were also found in other countries’ studies (Aladul et al., 2019; Teeple et al., 2019a; Teeple et al., 2019b). Regarding multiple switching between originators and biosimilars and substitution between biosimilars of the same generic name but with different brands, most respondents felt unsure or worried.

### Overall Findings in Current Practices of Biosimilars

This study demonstrated that biosimilars have not been widely utilized in China, as only 42.96% of HCPs had enrolled in the clinical practice of biosimilars, and fewer than one-third of patients had taken biosimilars. Among physicians, 57.58% had prescribed biosimilars. Fewer than half of them had experience with non-medical switching and prescribing biosimilars for unapproved indications. Although physicians are the main gatekeepers in determining biosimilar treatment for patients, patients’ awareness and consent are required for them before prescribing (McKinnon et al., 2018). Our study showed that most physicians (74/76, 97.37%) among those who had prescribed biosimilars had obtained consent from their patients when deciding the treatment regimen. Among pharmacists and nurses, practices related to biosimilars are insufficient, and further participation is required to ensure a better utilization of biosimilars. As the gaps in the level of knowledge and negative attitudes may trigger insufficient or even inappropriate practices in the uptake of biosimilars, which may also conversely aggravate the uncertainty and less-grounded attitudes (Rezk and Pieper, 2017), further evidence of biosimilars and training for stakeholders are of great importance to improve the practice of biosimilars.

According to HCPs’ response, the implementation of Therapeutic drug monitoring (TDM) and anti-drug antibody (ADA) test of biosimilars have not yet been adopted in real routine practice. The relative novelty, the inherent complexity of biosimilars and the inmaturity of the analysis methods might be several contributors to this phenomenon. Pharmacovigilance measures have been taken in a few HCPs’ departments, including developing risk management protocols, recording product names and batch numbers to ensure traceability and monitoring unexpected adverse events.

Healthcare regulatory practitioners play a key role in the whole lifecycle of biosimilars. Suggestions on the regulations of biosimilars could serve as references for improving the future development of biosimilars in China, such as updating the technical guidance for the development and evaluation of biosimilars, putting forward the concept of “interchangeable biosimilars” and setting regulations on the clinical switching of biosimilars in China.

### Recommendations for Promoting the Uptake of Biosimilars

Based on the results of our current study, several recommendations might be considered to fill the gap between the knowledge, attitude and uptake of biosimilars. As studies showed medical staff are the most trusted source of information about biosimilars for patients (Peyrin-Biroulet et al., 2017; Vandenplas et al., 2021a), it is necessary for HCPs to conduct patient education about biosimilars to promote uptake. Specific strategies for educating patients about biosimilars have already been proposed in some studies (Vandenplas et al., 2021b; Oskouei and Kusmierczyk, 2021). In addition, HCPs should enhance the understanding of biosimilars so that they can be well-armed to communicate adequately with patients. As experts in pharmacotherapy, clinical pharmacists could play a pivotal role in providing unbiased and up-to-date information for the medical team (Jarrett and Dingermann, 2015).

Efficacy, safety, and immunogenicity are key aspects that biosimilar-related stakeholders are concerned about. Evidence in real-world clinical practice needs to be accumulated, and HCPs are encouraged to take an active part in evidence-based evaluation to elucidate a comprehensive profile of biosimilars. An evidence-based guideline for clinical medication of biosimilars is now under development by our team (International Practice Guideline Registry Platform, 2020; Hu et al., 2021), which we hope to promote the appropriate utilization of biosimilars. In addition, TDM, a valuable tool for improving the efficacy and safety of biosimilars, should be further implemented in clinical medication (Chinese Pharmacological Society and China-Japan Friendship Hospital, 2020). In order to have the safety risks under control, recording the product names and batch numbers is suggested to ensure the traceability of biosimilars as well as their reference products.

Currently, perception of the interchangeability of biosimilars still needs to be improved among multiple stakeholders. Any decision to switch in clinical practice should be based on the patient’s individual condition as well as updated evidence-based research (Barbier et al., 2020) and expert consensuses (Kay et al., 2018; Gregory et al., 2020; Chinese Society of Clinical Oncology Anti-Lymphoma Alliance, 2021). Adequate monitoring of efficacy and safety is essential after switching to biosimilars. In addition, it is reported that nocebo effects associated with switching patients from originators to biosimilars can have unfavorable consequences for both patients and healthcare system, such as non-adherence and discontinuation of the treatment, substantial impaired quality of life, and the damage to the patient-clinician relationship (Kristensen et al., 2018). Thus, attention must be paid to the mitigation of a potential nocebo effect, and positive interaction between HCPs and patients is necessary (Colloca et al., 2019). Large-scale clinical studies with high quality on interchangeability are required as such research could pose a positive impact on biosimilar utilization (Jorgensen et al., 2017). Moreover, national recommendations and policies for interchangeability (including switching and substitution of biosimilars) are needed to support the uptake of biosimilars.

Extrapolation of indications in the realm of review and evaluation should be performed in full compliance with the local regulations. Regarding the medication of biosimilars for unapproved indications, from a Chinese perspective, biosimilars should only be extrapolated to unapproved indications after endorsement from the committee of pharmacy administration and pharmacotherapy in hospitals (Clinical Pharmacy Branch of Chinese Medical Association, 2020).

Building on other countries’ experience, similar to initiating chemical generics to treat patients with some chronic disease, biosimilars can also be encouraged to be included in the initial treatment regimen for naïve patients (Godman et al., 2021). Besides, several payment policies such as case-based payment, DRGs payment and DIP payment may set as incentives for promoting the uptake of biosimilars.

### Strengths of the Study

To the best of our knowledge, this present study is the first survey conducted in mainland China to investigate knowledge, attitudes and practices toward biosimilars among multiple stakeholders. The large sample size ensures the representativeness of respondents, especially healthcare providers. In addition, a comparison was conducted to determine the gap in knowledge and attitudes between different stakeholders. It is worth mentioning that, unlike most other studies with the same topic, our study recruited and analyzed healthcare regulatory practitioners specializing in drug regulation, medical insurance, medical administration and pharmacoeconomics for the first time in addition to HCPs and patients. Thus, this study depicted a comprehensive landscape of the current perspective on biosimilars among almost all stakeholders in China. Most importantly, with the results of identifying current attitudes and awareness of biosimilars in China, this study could provide a reference for promoting the uptake of biosimilars and ultimately reducing biologic therapy costs.

### Limitations and Future Perspectives

Several limitations should be considered for our study. First, it was not possible to calculate the response rate due to the unknown number of duplicate people in different WeChat groups. Second, this survey was an online voluntary study, and respondents who were willing to participate were mainly HCPs. Recruited healthcare regulatory practitioners and patients were in relatively small numbers, and thus, more sophisticated analysis was not suitable for these two groups. Further targeted studies for these two groups with larger sample sizes could be conducted to perform more precise analysis. In addition, most HCPs included in our study were oncologists and mainly from tertiary care centers. Future studies could be designed for a more specific comparison of different subgroups among HCPs in different specialties or different classes of medical institutes.

## Conclusion

This study revealed the current knowledge, attitudes and practice toward biosimilars among multiple stakeholders in mainland China. Healthcare providers and healthcare regulatory practitioners have a better understanding of biosimilars than patients, but there is still room for knowledge improvement regarding the development process and related regulations. Although stakeholders broadly recognize biosimilars important as a cost-saving measure, hindering factors of biosimilars uptake still exist, including concerns about efficacy, safety, immunogenicity, interchangeability and indication extrapolation of biosimilars. High-quality real world evidence, educational programs and other effective measures directed towards barriers as well as detailed regulations are still warranted to improve biosimilar utilization in China in the future.

## Data Availability

The original contributions presented in the study are included in the article/supplementary material, further inquiries can be directed to the corresponding author.
